# Molecular footprints of selection effects and whole genome duplication (WGD) events in three blueberry species: detected by transcriptome dataset

**DOI:** 10.1186/s12870-020-02461-w

**Published:** 2020-06-03

**Authors:** Yunsheng Wang, Fei Nie, Muhammad Qasim Shahid, Faheem Shehzad Baloch

**Affiliations:** 1 College of Health and Life Science, Kaili University, Kaili City, 556011 Guizhou Province China; 2 Biological institute of Guizhou Province, Guiyang City, 556000 Guizhou Province China; 3 State Key Laboratory for Conservation and Utilization of Subtropical Agro-Bioresources, South China Agricultural University, Guangzhou, 510642 China; 4 Guangdong Provincial Key Laboratory of Plant Molecular Breeding, South China Agricultural University, Guangzhou, 510642 China; 5 College of Agriculture, South China Agricultural University, Guangzhou, 510642 Guangdong Province China; 6 Department of Field Crops, Faculty of Agricultural and Natural Sciences, Abant İzzet Baysal University, Bolu, Turkey

**Keywords:** Adaptive evolution, Phylogenetic analysis, Polyploid, Unigene, *Vaccinium*

## Abstract

**Background:**

Both selection effects and whole genome duplication played very important roles in plant speciation and evolution, and to decipher the corresponding molecular footprint has always been a central task of geneticists. *Vaccinium* is species rich genus that comprised of about 450 species, and blueberry is one of the most important species of *Vaccinium* genus, which is gaining popularity because of high healthful value. In this article, we aimed to decipher the molecular footprints of natural selection on the single copy genes and WGD events occur in the evolutionary history of blueberry species.

**Results:**

We identified 30,143, 29,922 and 28,891 putative protein coding sequences from 45,535, 42,914 and 43,630 unigenes assembled from the leaves’ transcriptome assembly of 19 rabbiteye (T1), 13 southern highbush (T2) and 22 northern highbush (T3) blueberry cultivars. A total of 17, 21 and 27 single copy orthologs were found to undergone positive selection in T1 versus T2, T1 versus T3, and T2 versus T3, respectively, and these orthologs were enriched in metabolic pathways including “Terpenoid backbone biosynthesis”, “Valine, leucine and isoleucine biosynthesis”, “Butanoate metabolism”, “C5-Branched dibasic acid metabolism” “Pantothenate and CoA biosynthesis”. We also detected significant molecular footprints of a recent (about 9.04 MYA), medium (about 43.44 MYA) and an ancient (about 116.39 MYA) WGD events that occurred in the evolutionary history of three blueberry species.

**Conclusion:**

Some important functional genes revealed positive selection effect in blueberry. At least three rounds of WGD events were detected in the evolutionary history of blueberry species. Our work provides insights about the genetic mechanism of adaptive evolution in blueberry and species radiation of *Vaccinium* in short geological scale time.

## Background

Mutation-selection process is the most fundamental mechanism of evolution, the mutations are the source on which natural selection operates and eventually natural selection lead to an optimization process of allele (mutant) frequencies [1]. Identifying alleles or genes influenced by natural selection have fascinated the geneticists for a long time [2, 3]. So far, more than ten statistical methods have been introduced to detect the molecular footprints that resulted in natural selection [4, 5]. Of these, the method of Ka/Ks ratio (Ka: the number of non-synonymous substitutions per non-synonymous site, and Ks: the number of synonymous nucleotide substitutions per synonymous site) has a great impact on estimation of selection pressure and understanding the evolutionary dynamics of protein-coding sequences across closely related and yet diverged species [6–8].

Whole genome duplication (WGD) or polyploidization event is another evolutionary genetic phenomenon that has long fascinated geneticists for its great significance in speciation and evolution [9–12]. For example, WGD event is an important force for the species radiations of many plant lineages [13–17]. According to the age and outcomes of cytogenetics and karyotype, WGD events can be classified into two kinds, one is the recent or new polyploidization event, which results in “autopolyploids” and emerge from the fusion of gametes produced by non-meiosis, or hybridization of normal meiosis gametes of same species individuals, or “allopolyploids” produced from inter-specific hybridization [18]. About 47–70% of angiosperm species are estimated as polyploid and approximately 25% of vascular plants have undergone recent polyploidization [19, 20], and this WGD event could be detected by karyotyping and flow cytometry method [21]. Another is the ancient polyploidization event, which happened in earlier geological ages. It is believed that all existing angiosperms are paleopolyploids, and have undergone two or more rounds of ancient polyploidization events in their evolutionary history [22, 23]. In spite of subsequent diploidization (massive gene loss and structural rearrangements) or fractionation events occur and made the polyploidy return to a diploid state, the footprint of ancient WGD would still be detected in genome sequence in the form of blocks of duplicated genes [24, 25].

High-throughput genome data produced by next-generation sequencing technology is being used frequently to investigate the key issues of evolutionary genetics [26–28]. However, full genome sequencing remained impractical for some non-model species with polyploid genome, high heterozygosity or high GC contents in the genome. In such cases, transcriptome sequencing provides ideal alternative with the advantages including sequencing cost, rich in information, coding protein, functional prediction, and no species or individual restrictions [29–31]. The transcriptome sequencing has been widely used for detection of molecular footprints of evolutionary studies including selection effect and WGD in recent years [32–36].

*Vaccinium* is a young and widespread genus in Ericaceae, and contains about 450 species [37, 38]. The species number of *Vaccinium* genus is far more than average species number in plant at genus level (about 67 per genus) and in Ericaceae (about 80 per genus) (http://www.theplantlist.org/statistics/). Obviously, the *Vaccinium* species had undergone a recent and evolutionary radiation. In recent decades, the *Vaccinium* species have attracted more and more attentions for high antioxidant contents in fruit of many species (e.g. blueberries, cranberries, bilberry, and lingonberry) that have nutritional and therapeutic effects [39]**.** Blueberry is one of the most important flora of *Vaccinium*, which is composed of about 20 species [38]. Beside six diploid species, all blueberry species are polyploid, and some have complex polyploid genome, such as *Vaccinium corymbosum* [40]. Despite of only one century history of domestication and cultivation, blueberry has been listed in the top five economically important, non-citrus fruits in North America [41, 42]. The main reason for the popularity of blueberry is its excellent dietary therapy functions that produced from high anthocyanins contents in fruits [30, 43–45]. The current commercially planting blueberry varieties are principally derived from diploid lowbush blueberry (*V. myrtilloides*), tetraploid lowbush blueberry (*V. angustifolium*), northern tetraploid highbush blueberry (*V. corymbosum*), hexaploid rabbiteye blueberry (*V. virgatum*), southern highbush blueberry (hybrid from *V. corymbosum L* × native southern species) and semi-highbush blueberry (hybrid from *V. corymbosum* × *V. angustifolium*) [46, 47]. Among them, rabbiteye, northern highbush, and southern highbush blueberries are widely planted at commercial level.

Until now, like many important crops, blueberry research has entered the genome era. For example, the genome draft of a northern high-bush blueberry has been published [48, 49]. The transcriptome analyses have been used for blueberry studies about characteristics of gene expression in the cold environment [50–52], the metabolics related genes of blueberry antioxidant substances [53], the changes of gene expression profiles in blueberry after infection with *Colletotrichum acutatum* [54], the gene expression dynamics during five blueberry fruit development stages [48], and detection of molecular markers [55]. However, the reports about the genome evolution of blueberry are limited. In this article, we analyzed the assembled transcriptome data, which was generated from second generation sequencing platform, and the major aims were to (1) re-construct the phylogenetic relationship among 54 blueberry cultivars, (2) identify the genes undergone selection effect, (3) and to detect the WGD event in the evolutionary history of blueberry, and provide insights on the genetic mechanism about evolutionary radiation of *Vaccinium* species.

## Results

### Summary of protein coding unigenes

From 45,535 assembled unigenes in rabbiteye blueberry (T1), 42,914 in southern highbush blueberry (T2) and 43,630 in northern highbush blueberry (T3) species, we identified 30,143, 29,922 and 28,891 protein coding unigenes according to the annotation of Nr, Swiss-Prot, GO and KEGG databases, and by using program ESTScan in three blueberry species, respectively (Table 1).

**Table 1:** The number of protein coding unigenes detected from the total assembled transcripts of 54 cultivars of three blueberry species

Code	Total number of transcripts	Total number of transcripts annotated by at least one of Nr, Swiss-Prot, GO and KEGG	Total number of transcripts predicted by ESTScan	Total number of protein coding transcript
T1	45,535	28,091	2052	30,143
T2	42,914	28,115	1807	29,922
T3	43,630	27,256	1635	28,891

T1, T2 and T3 indicate rabbiteye (*V. virgatum*), southern highbush (*V. corymbosum* × southern species), and northern highbush blueberry (*V. corymbosum*) species, respectively

### Gene families’ statistics

**Fig. 1:**
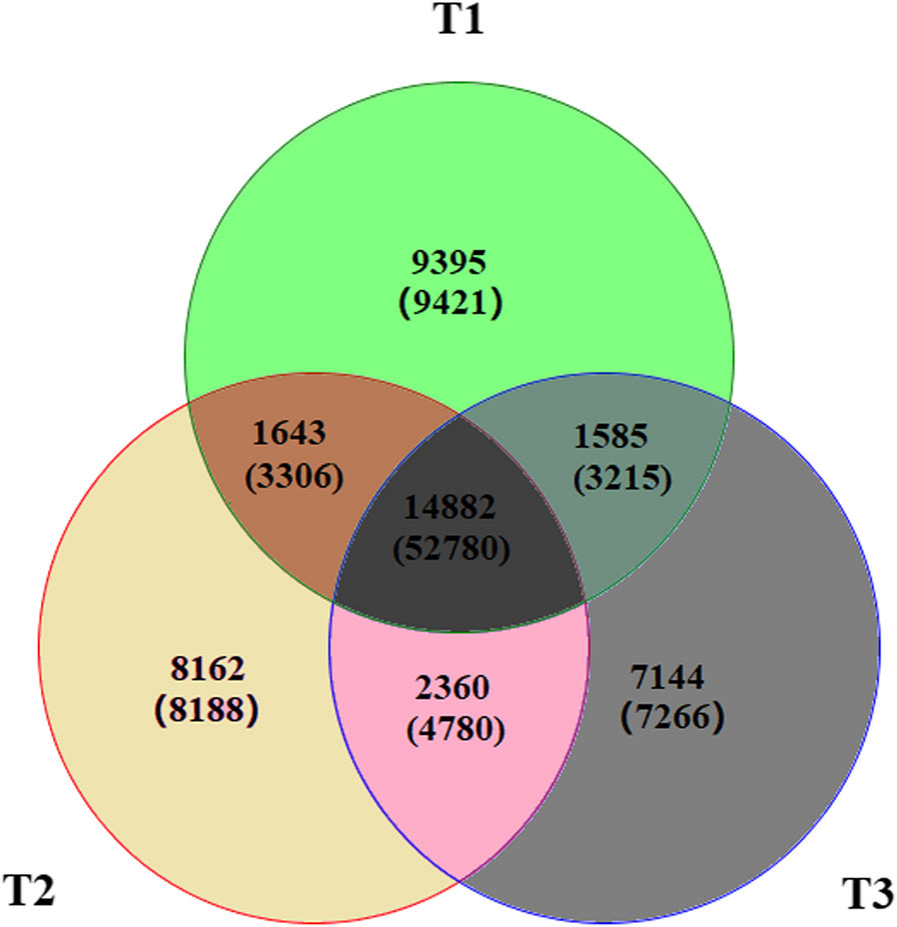
Venn analysis of gene families in three blueberry species. T1, T2 and T3 indicate rabbiteye (*V. virgatum*), southern highbush (*V. corymbosum* × southern species), and northern highbush blueberry (*V. corymbosum*) species, respectively. The numbers without parenthesis represent the gene families, and the number in parenthesis represent the genes number

We detected 45,171 genes families from protein coding unigenes of three blueberry transcriptomes. Of these, 14,882 gene families, including 52,780 unigenes, were shared by all three blueberry transcriptomes. In total, 1643 gene families, including 3306 unigenes were shared between rabbiteye and southern highbush blueberry, 2360 gene families including 4780 unigenes were shared by southern highbush and northern highbush blueberry, and 1585 gene families including 3215 unigenes were common between rabbiteye and northern highbush blueberry. A total of 9395 gene families including 9421 unigenes, 8162 gene families including 8188 unigenes, and 7144 gene families including 7266 unigenes were specific to rabbiteye, southern highbush and northern highbush blueberry species, respectively. We also identified 12,688 homologous single copy gene families (orthologs) and 2915 multi-copy gene families shared by all three blueberry species (Table 2; Fig. 1).

**Table 2:** Summary of gene families detected in three blueberry species

Species	Coding of protein gene	Gene families	Specific gene	Single copy gene families	Multi-copy homologous genes
T1	30,143	9395	9421	9371	24
T2	29,922	8162	8188	8136	26
T3	28,891	7144	7266	7028	115
T1,T2	NA	1643	3306	1625	18
T1,T3	NA	1585	3215	1546	39
T2,T3	NA	2360	4780	2308	52
T1,T2,T3	NA	14,882	52,780	12,688	2195
Total	88,956	45,171	88,956	42,702	2469

T1, T2 and T3 indicate rabbiteye (*V. virgatum*), southern highbush (*V. corymbosum* × southern species), and northern highbush blueberry (*V. corymbosum*) species, respectively

*NA* Not applicable

### Genetic relationships among blueberry cultivars

**Fig. 2:**
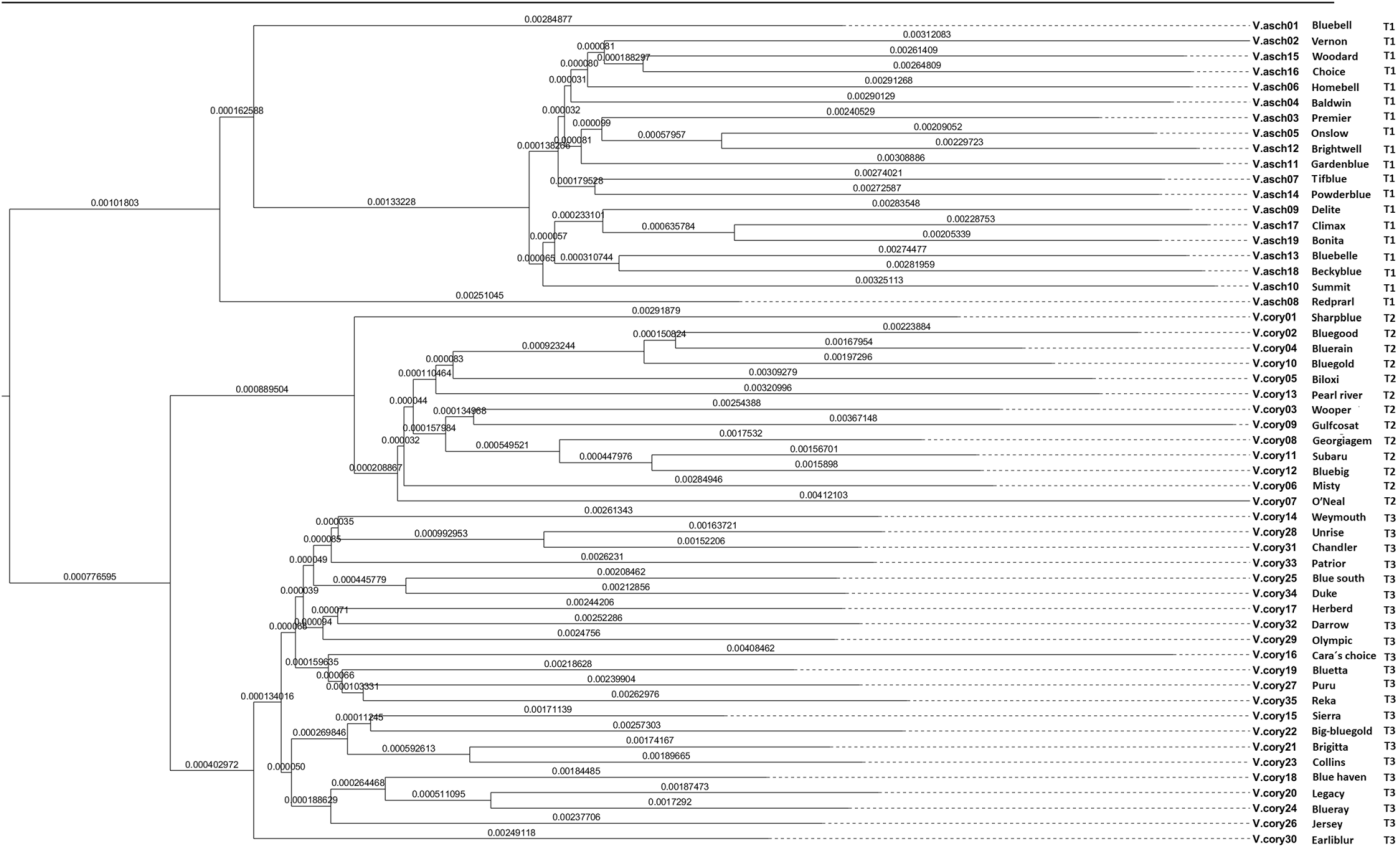
Phylogenetic tree of 54 blueberry cultivars constructed by single copy unigenes. T1, T2 and T3 indicate rabbiteye (*V. virgatum*), southern highbush (*V. corymbosum* × southern species) and northern highbush blueberry (*V. corymbosum*) species, respectively

In the phylogenetic tree, all the cultivars clustered into two main groups, the rabbiteye cultivars grouped into a main group, and southern highbush and northern highbush blueberry cultivars clustered into another main group, and then separated into two large groups having same kind of blueberry in a group. In rabbiteye group, which was comprised of 19 cultivars, the sample V.asch01 (Cultivar:Bluebell) and V.asch08 (Cultivar:Summit) were far away from other cultivars in the genetic distance or phylogenetic relationship, and V.asch09 (Cultivar:Delite), V.asch17 (Cultivar:Climax), V.asch19 (Cultivar:Bonita), V.asch13 (Cultivars Bluebelle), V.asch 18 (Cultivar:Beckyblue) were relatively close, and the other cultivars have relatively close phylogenetic relationship with each other. In the southern highbush group of 13 cultivars, V.cory01 (Cultivar:Sharpblue), V.cory07 (Cultivar:O′Neal) and V.cory06 (Cultivar:Misty) located at the bottom of the phylogenetic tree, and showed a farther kinship. In the northern highbush group of 22 cultivars, the V.cory30 (Cultivar:Earliblur) was located at the bottom of cluster, and V.cory15 (Cultivar:Sierra), V.cory22 (Cultivar:Big-bluegold), V.cory21 (Cultivar:Brigitta), V.cory23 (Cultivar:Collins), V.cory18 (Cultivar:Bluehaven), V.cory20 (Cultivar:Legacy), V.cory24 (Cultivar: Blueray) and V.cory26 (Cultivar: Jersey) clustered into a major branch with relatively close affinity, and all other cultivars assembled into another major branch, which have relatively close phylogenetic relationship (Fig. 2).

### Selection effect of single copy homologous unigenes

**Fig. 3:**
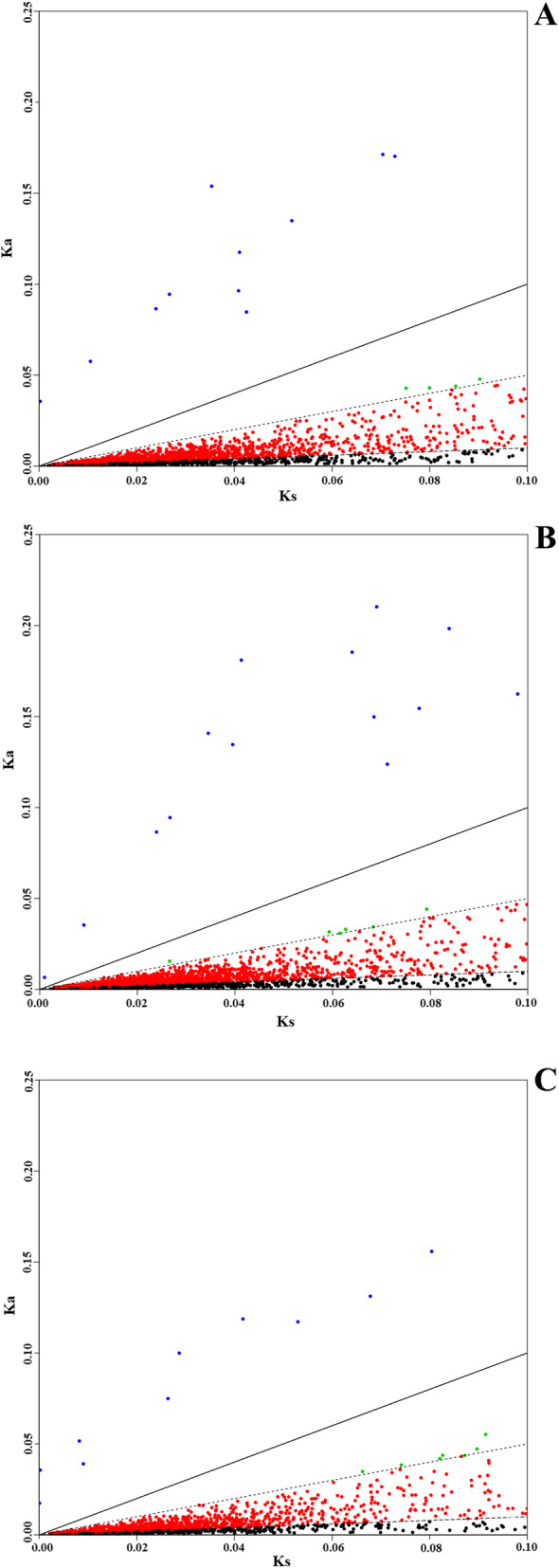
Distribution of Ka/Ks values of single copy orthologs between three blueberry species. **a***V. virgatum* Vs *V. corymbosum*; **b***V. virgatum* Vs (*V. corymbosum* × southern species); **c**: *V. virgatum* Vs (*V. corymbosum* × southern species). X-axis indicates the value of Ka (Nonsynonymous substitution rate), Y-axis indicates the value of Ks (synonymous substitution rate) of single copy orthologs. Blue plots represent the single copy orthologs with Ka/Ks more than 1; green plots represent the single copy orthologs with Ka/Ks between 0.5 and 1; red plot represent the single copy orthologs with Ka/Ks between 0.1 and 0.5; black plots represent the single copy orthologs with Ka/Ks less than 0.1

**Fig. 4:**
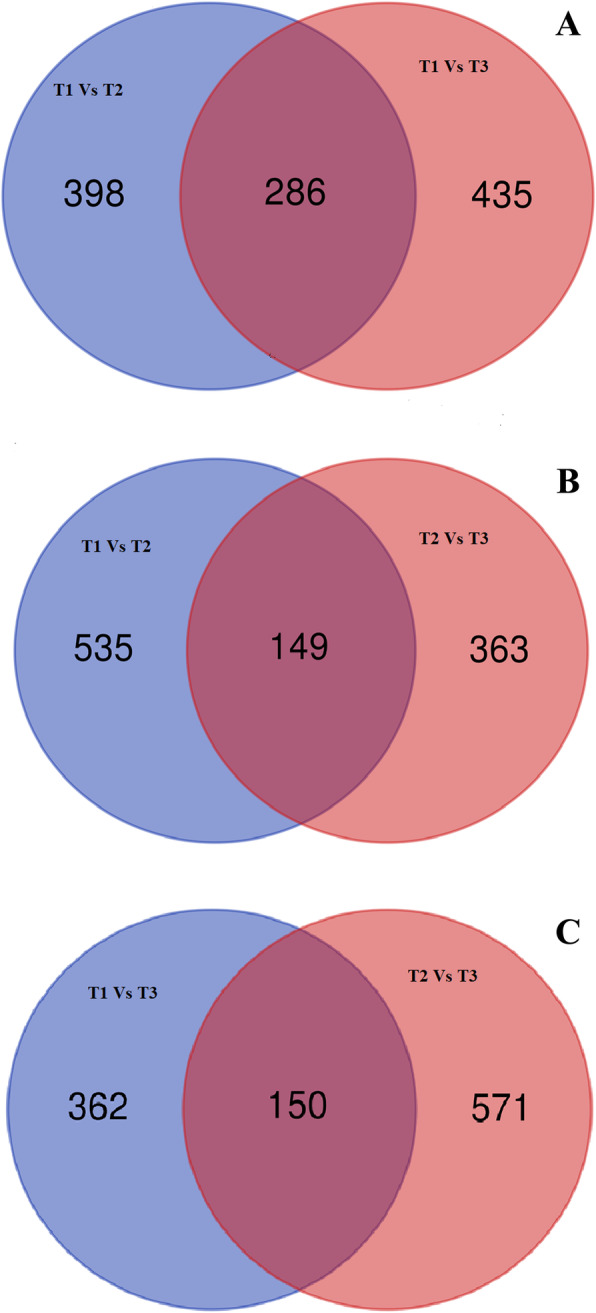
Venn analysis of unigenes undergone purifying selection in three blueberry species. T1, T2 and T3 indicate rabbiteye (*V. virgatum*), southern highbush (*V. corymbosum* × southern species) and northern highbush blueberry (*V. corymbosum*) species, respectively

Of the 12,688 single copy orthologs in three blueberry species, 2276, 2452, 1711 orthologs were detected in T1 versus T2, T1 versus T3 and T2 versus T3 after filtering with Ks > 0.1 and applicable Ka and Ks. Among them, 13, 15 and 10 orthologs showed significant footprints of strong positive selection (Ka/Ks > 1); 4, 6 and 7 orthologs Ka/Ks values were 0.5 < Ka/Ks < 1, which displayed the footprints of weak positive selection; 684, 721 and 512 orthologs Ka/Ks showed the footprints of purifying selection (Ka/Ks < 0.1); 1575, 1710 and 1180 orthologs exhibited neutral evolution with Ka/Ks between 0.1 ~ 0.5 in three groups (Table 3; Fig. 3; Table S1). In total, 32 unigenes in T1, 31 unigenes in T2 and 37 unigenes in T3 were identified to undergo positive selection (Table 4; Table S2). Of these positive selective unigenes, nine unigenes in T1 produced 91 GO terms (62 GO terms were associated with biological process, 18 GO terms with cellular component and 11 GO terms with molecular function). Among them, four GO terms (GO:0016772, GO:0010506, GO:0031329 and GO:0009894) were found to be significantly enriched. A total of 10 unigenes in T2 produced 185 GO terms (98 GO terms were related to biological process, 39 GO terms with cellular component and 48 GO terms with molecular function). Of these, 8 GO terms (GO:0010506, GO:0031329, GO:0009894, GO:0010506, GO:0031329, GO:0016485, GO:0051604 and GO:0009894) were found to be significantly enriched. Fourteen unigenes in T3 produced 234 GO terms (135 GO terms were associated with biological process, 38 GO terms with cellular component and 61 GO terms with molecular function). Among them, six GO terms (GO:0016744, GO:0010506, GO:0031329, GO:0009894, GO:0016485 and GO:0051604) were found to be significantly enriched (Table 4 Table S3). Of positive selective unigenes, eight unigenes were annotated by KEGG database, T1-Unigene0035236 and T3-Unigene0033665, T2-Unigene0000660 and T3-Unigene0000319, T2-Unigene0021375 and T3-Unigene0020145, and T2-Unigene0023454 and T3-Unigene002102 were orthologous pairs. Among them, T2-Unigene0023454 and T3-Unigene002102 were the putative gene encoding “Ste24 endopeptidase” whose ID was 3.4.24.84 in KEGG database, and “Ste24 endopeptidase” is a key enzyme involving the metabolic pathway of “Terpenoid backbone biosynthesis” whose ID was ko00900 in KEGG. T1-Unigene0035236 and T3-Unigene0033665 were the putative genes encoding “Acetolactate synthase” whose ID was 2.1.1.6 in KEGG database, and Acetolactate synthase is a key enzyme of multi-pathways such as “Valine, leucine and isoleucine biosynthesis (Ko00290)”, “Butanoate metabolism (Ko00650)”, “C5-Branched dibasic acid metabolism (Ko00660)” “Pantothenate and CoA biosynthesis (Ko00770)”, and these pathways were hit by above two orthologous pairs and found to be significantly enriched (Table 4; Table S4). A total of 1119, 1047 and 1083 unigenes in T1, T2 and T3 were detected to undergo purifying selection by at least orthologous of one groups (T1 vs T2, T1 vs T3 and T2 vs T3), of which, 286, 149 and 150 unigenes in T1, T2 and T3 were found to be overlapped between T1 vs T2 and T1 vs T3 (Fig. 4a), T1 vs T2 and T2 vs T3 (Fig. 4b), and T1 vs T3 and T2 vs T3 (Fig. 4c), respectively.

**Table 3:** Ka/Ks test statistics of single copy orthologs number in three blueberry species

Species 1	Species 2	Total single copy Orthologous pairs	Filtered Orthologous pairs with Ks > 0.1 and applicable Ks or Ka	Orthologous pairs with Ka/Ks > 1	Orthologous pairs with 0.5 > Ka/Ks > 1	Orthologous pairs with 0.1 > Ka/Ks > 0.5	Orthologous pairs with Ka/Ks < 0.1
T1	T2	12,688	2276	13	4	1575	684
T1	T3	12,688	2452	15	6	1710	721
T2	T3	12,688	1711	10	7	1180	512

T1, T2 and T3 indicate rabbiteye (*V. virgatum*), southern highbush (*V. corymbosum* × southern species), and northern highbush blueberry (*V. corymbosum*) species, respectively

**Table 4:** Statistics of unigenes undergone positive selection effect in three blueberry species

Species	Intensity of positive	Species pairs	Number of unigenes	Number of unigenes annotated by GO database	Number of GO terms	Enriched Go term (*p* < 0.05)	KEGG annotation	Enrich KEGG (*p* < 0.05)	Ko ID [Enzyme ID]
T1	Strong	T1 Vs T2	13	1	4(4 M)	ND	0	0	ND
		T1 Vs T3	15	3	5(5 M)	GO:0016772	0	0	ND
		Shared	6	1	0	ND	0	0	ND
	Weak	T1 Vs T2	4	3	16(3C + 2 M + 11B)	GO:0010506; GO:0031329; GO:0009894	0	0	ND
		T1 Vs T3	6	4	66(15C + 51B)	ND	2	1	ko00660; ko00650; ko00290; ko00770; ko01210; ko01230 [2.2.1.6]
		Shared	1	0	0	ND	0	0	ND
T2	Strong	T1 Vs T2	13	1	4(4 M)	ND	0	0	ND
		T2 Vs T3	10	4	50(11 M + 31B + 8C)	ND	2	0	ND
		Shared	2	0	0	ND	0		ND
	Weak	T1 Vs T2	4	3	35(14C + 8 M + 13B)	GO:0010506; GO:0031329; GO:0009894	0	0	ND
		T2 Vs T3	7	5	96(17C + 25 M + 54B)	GO:0010506;GO:0031329;GO:0016485;GO:0051604;GO:0009894		1	ko00900 [3.4.24.84]
		Shared	1	1	0	ND	0	0	ND
T3	Strong	T1 Vs T3	15	3	5(5 M)	ND	0	0	ND
		T2 Vs T3	10	4	51(8C + 12 M + 31B)	ND	2	0	ND
		Shared	1	0	0	ND	0	0	ND
	Weak	T1 Vs T3	6	4	88(15C + 23 M + 50B)	GO:0016744	2	1	ko00660; ko00650; ko00290 ko00770; ko01210; ko01230 [2.2.1.6]
		T2 Vs T3	7	5	90(15C + 21 M + 54B)	GO:0010506; GO:0031329; GO:0009894; GO:0016485; GO:0051604	1	1	ko00900 [3.4.24.84]
		Shared	0	0	0	ND	0	0	ND

*ND* indicates no data; M represents “molecular function”; C represents “cellular component”; B represents “Biological process”

T1, T2 and T3 indicate rabbiteye (*V. virgatum*), southern highbush (*V. corymbosum* × southern species), and northern highbush blueberry (*V. corymbosum*) species, respectively

### Genome wide duplication events of three blueberry species

**Fig. 5:**
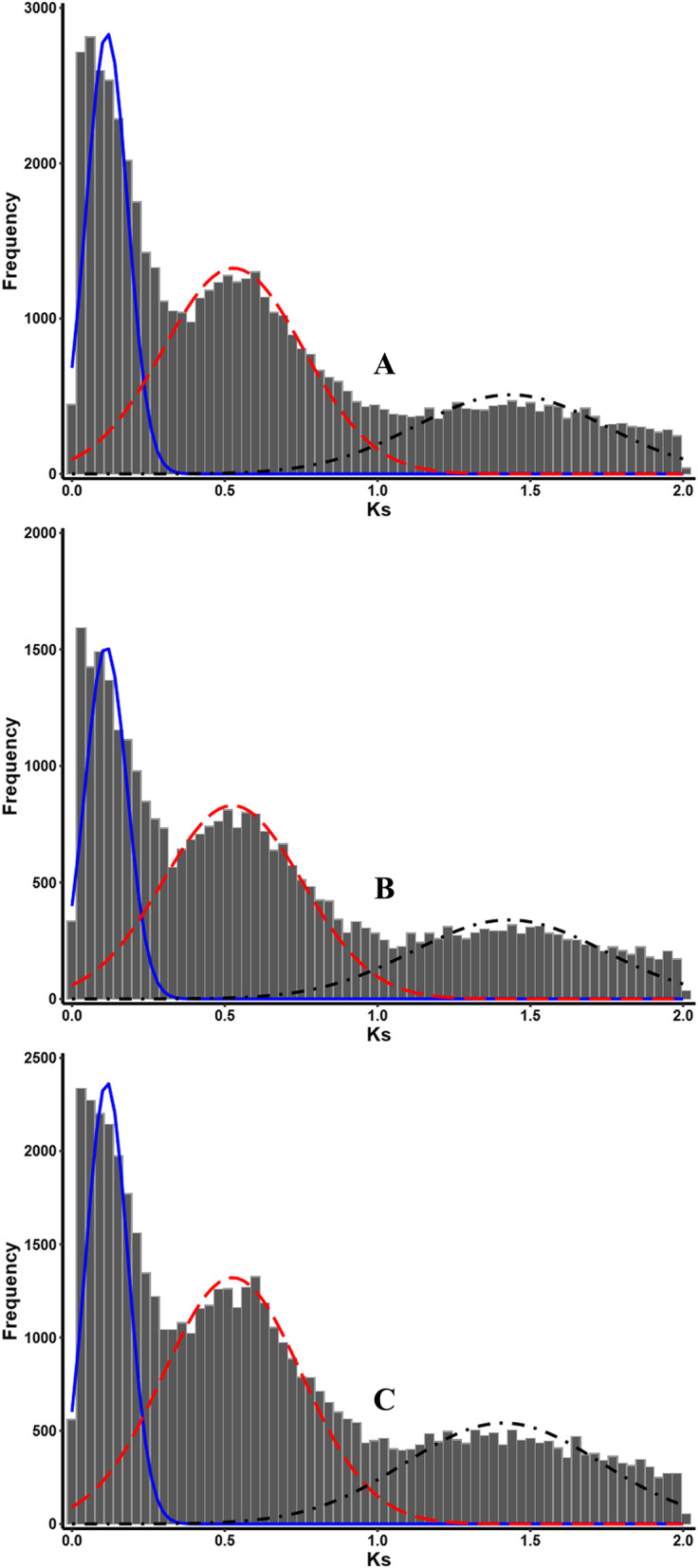
Ks age distribution of paralogs in three blueberry species. **a***V. virgatum*; **b** (*V. corymbosum* × southern species) *V. corymbosum*; **c***V. corymbosum*. X-axis indicates the Ks (synonymous substitution rate) value of paralogous, Y-axis indicates the number of paralogous for a specific Ks value range

Ks age distribution of paralogs in T1, T2 and T3 displayed three significant peaks conforming to the normal distribution with almost similar fitting curve. The first peak in three blueberry species were detected with a mode at Ks ≈ 0.11, and it represented a recent shared WGD event that happened about 9.02 million years ago in the evolutionary history of three kinds of blueberry species. The second peak with a mode at Ks ≈ 0.53 in three blueberry species represented a medium shared WGD event that happened in the evolutionary history of three kinds of blueberry species about 43.44 million years ago. The third peak with a mode near at Ks ≈ 1.42 in three blueberry species indicated an ancient shared WGD event in the evolutionary history of three different species of blueberry about 116.39 million years ago. Among three peaks, the recent peak of Ks age distribution was thinnest for the smallest variability of normal distribution, while the third one was the thickest for the biggest variability of normal distribution, and this trend was in accordance with the evolutionary dynamics of subsequent diploidization of WGD events (Fig. 5, Table 5).

**Table 5:** The average value (μ) and standard deviation (σ) of normal distribution peaks of fitted curve based on Ks age distribution

Species	The recent peak	The median peak	The oldest peak
T1	0.1144 ± 0.0678	0.5281 ± 0.2294	1.4347 ± 0.3103
T2	0.1113 ± 0.0681	0.5250 ± 0.2283	1.4298 ± 0.31298
T3	0.1138 ± 0.0687	0.5255 ± 0.2270	1.4118 ± 0.32068

T1, T2 and T3 indicate rabbiteye (*V. virgatum*), southern highbush (*V. corymbosum* × southern species), and northern highbush blueberry (*V. corymbosum*) species, respectively

## Discussion

### Genetic relationships among blueberry cultivars

Clarifying the genetic or phylogenetic relationships among organisms are greatly helpful for parent selection in breeding projects [56]. In the last three decades, the molecular markers such as RAPD, AFLP, SSR, SNP have played an important role to study the phylogenetic relationship of individuals among intra- and inter-species [57, 58]. In the genome era, large scale SNP data have been developed by using next-generation high throughput sequencing technologies at unprecedented speed [59, 60], and this makes SNP markers the most frequent and convenient tool to study phylogenetic relationship of organisms [61, 62]. Besides above molecular markers, the single copy orthologues genes also regarded as excellent markers to study the phylogenetic relationship of diploid or polyploid individuals between intra- and inter-species [63, 64]. The sources of blueberry cultivars are complex, so clarifications by phylogenetic relationships are meaningful for the identification, protection and breeding of blueberry germplasm. Previous studies have used different molecular makers, including RAPD, SSR and SNP as a tool to construct the phylogenetic relationship of blueberry germplasm with different genetic origin [65–69]. In spite of the blueberry cultivars or breeding materials are from different origins or species with different ploidy levels, the SSR and SNPs marker seems to be an effective tool to differentiate and categorize blueberry cultivars [65, 67, 69]. Here, we used single copy nuclear gene dataset to reconstruct the phylogenetic relationships of three blueberry species, and detected a good differentiation and grouping of blueberry cultivars with different origins.

### Selection effects on single copy genes of T1, T2 and T3

Whole genome duplication events occurred very commonly during the evolution of flowering plants [70]. However, some genes would return to single copy statue after suffering the duplication events, because one copy would lose randomly in the genome by genetic drift or duplication-resistant by dosage-sensitive selection in the next evolutionary process [71, 72]. In the recent years, single copy genes have attracted many researchers because of excellent application in genetics, for example, single copy gene is a good molecular tool to construct the phylogenetic tree of species [73]. Logically, without duplication copy, the single copy homologous genes could be regarded as orthologs, and paralogs don’t disturb the evolutionary analysis, which made the single copy genes as the merit research object for the molecular mechanism of selection effect and speciation of organism [27, 34, 36, 55, 64, 73, 74]. In this study, we also only test the single copy unigenes to identify the molecular footprints of selection effects by using the method of Ka/Ks. In evolutionary genetics, Ks indicates synonymous substitute rate, and Ka indicates non-synonymous substitute rate of protein coding genes. It is generally presumed that synonymous mutations are not subjected to natural selection, while non-synonymous mutations would be subjected to natural selection for the change of structure and function led by the amino acid composition change in a coding protein [75]. Ka/Ks indicates the ratio of mutations that change a specific protein structure and do not change a specific protein, and the value gives a clear idea to judge the evolutionary dynamics of orthologous protein-coding sequences across closely related and yet diverged species [76, 77]. Theoretically, it is thought that Ka/Ks = 1 indicates the protein coding genes under neutral evolution, Ka/Ks > 1 represents more non-synonymous substitutions occurred in orthologs, and suggesting that the genes evolved under positive selection, and Ka/Ks < 1 indicates that the deleterious substitutions are eliminated by purifying selection (negative selection). But in empirical research, most genes with Ka/Ks > 0.5 have undergone adaptive evolution by combining with Tajima’test, and Fu & Li` test, and so the threshold of Ka/Ks > 0.5 was always regarded as positive selection [78–81]. In this study, we followed the previous criteria and set 1 > Ka/Ks > 0.5 as weak positive selection, and Ka/Ks > 1 as strong positive selection. We also filtered the candidate orthologs with Ks value > 0.1, as these may be paralogs as described by Zhang et al. [78] and Zhou et al. [82]. Our results revealed that most of the single copy unigenes evolved through neutral pattern. Besides, some single copy unigenes undergone negative selection, and their numbers were far more than that undergone positive selection (Table S2). This result illustrated that the occurrence of harmful mutations is more frequent than that of beneficial mutations in blueberry genome. Among unigenes undergone positive selection effect, we identified one ortholog (T2-Unigene0000660 and T3-Unigene0000319) that encoded “disease resistance protein RPS2”, and involved into metabolic pathway “Plant-pathogen interaction (ko04626)” (Table S4). We speculated that this ortholog is a merit candidate gene for the breeding and improvement of blueberry cultivars and their ecological adaptation. According to our knowledge, this is the first report about the blueberry genome or gene selection effect in three species.

### Whole genome duplication in evolutionary history of blueberry

The advent of plant genomics era has revealed the importance and ubiquity of WGD or polyploidy in the evolutionary history of species [83], because ancient WGD events could be conveniently and accurately reconstructed by using Ks age distribution analysis, or by detecting and comparative analysis of the collinear regions of intra- and inter-genome, or by constructing the molecular phylogenetic tree based on high throughput genome data [84]. Ks age distribution analysis has some significant merits including relatively low computational cost, available in the limited part of paranome, e.g. based on EST collections and no requirement of positional information on the paralogs, and was used frequently in the detection of WGD events [85]. Therefore, in this study, we also used Ks age distribution analysis to deduce the WGD event in the evolutionary history of T1, T2 and T3.

Ren et al. (2018) [35] detected the widely WGD events by using 105 genome/transcriptome data of different seed plant species including *V. corymbosum*, and made a conclusion that WGDs are commonly found in species-rich lineages of eukaryotes. However, they did not find WGD events in *V. corymbosum* based on the transcriptome data. *V. corymbosum* (T2) are polyploid species as well as T1 and T3 [40], so the recent or new WGD events in the evolution history of T1, T2 and T3 should be detected. Colle et al. (2019) [49] deduced that a very recent polyploidization event occur in *V. corymbosum* approximately 69 to 77 thousand years ago based on the Ks divergence between homologs in highbush blueberry and the pair-wise LTR difference of 0.18–0.20% between the four haplotypes. However, whether this polyploidization event shared by other blueberry species is still unknown. In this study, even though the Ks age distribution histogram were not completely consistent from different transcriptome of three blueberry species, we detected three significant peaks with normal distribution in three blueberry species. Based on the fitting normal distribution curve, the median Ks value and standard deviation of first peak in T1, T2 and T3 were almost same, and similar trends were followed by second and third peaks. So, according to the histogram of Ks age distribution constructed by each paralogs dataset of T1, T2 and T3, we deduced that at least three rounds of WGD events occur in the evolutionary history of blueberry species. The recent WGD event was found to occur approximately 9.04 million years ago before differentiation of three blueberry species, even might be before the differentiation of section Cyanococcus, which included about 20 species and most of them are polyploid, because all three WGD event shared by three species, and this WGD event might have played an important role in the speciation and evolution of Cyanococcus species. *Vaccinium* is species rich genus that comprised of about 450 species [38]. The polyploidization event is the important dynamics for plant species diversification [11, 86]. So, the second WGD event, about 43.44 million years old, may have contributed greatly to the speciation of genus *Vaccinium*. In this study, we also detected an ancient WGD event about 116.39 million years old, and this was in accordance with the paleohexaploidization event (γ) shared by the eudicots [86, 87]. The difference in median Ks value between three blueberry species and other species can be explained by different substitution rates among the different plant lineages [86, 88, 89]. The results offer clear molecular footprints of three rounds of WGD events that occurred in the history of blueberry species, and it is helpful for understanding the molecular mechanism of complex evolutionary of blueberry species, even linages of Ericaceae.

## Conclusion

Single copy nuclear gene dataset displayed a good differentiation and grouping of blueberry cultivars with different origins. Some important functional genes undergone positive selection in blueberry, and showed an adaptive evolution pattern. At least three rounds of WGD events were detected in the evolutionary history of blueberry species. Our work provides insights about the genetic mechanism of adaptive evolution in blueberry and species radiation of *Vaccinium* in short geological scale time.

## Methods

### Identification of putative protein coding sequences from blueberry transcriptome

A total of 45,535, 42,914 and 43,630 unigenes assembled from the leave’s transcriptomes of 19 rabbiteye, 13 southern highbush and 22 northern highbush blueberry cultivars (Accession ID in Genbank: PRJNA511922) were analyzed as a basic data in this article. The samples information, construction of library, sequencing and assembling of unigenes had been published and described previously [55]. In short, 2–3 years old seedlings of 54 blueberry cultivars (information listed in Fig.2) were collected from blueberry germplasm nursery of Majiang Blueberry Industry Engineering Technology Center (Wuyangma village, Xuanwei town, Majiang county, Guizhou province, China), and total RNA was extracted from young leaves by total RNA kit (STRN50-1KT|Sigma) and strictly followed the guidelines provided by the company. The RNA-sequencing libraries were prepared as follow: Firstly, the total RNA was treated with DNAse and then poly-T-oligo-attached magnetic beads was used to filter poly-A-containing mRNA from the total RNA. Secondly, about 300 ~ 500 base length fragments were obtained from the purified mRNA sequences, and the first single strand of cDNA was produced by using these fragments as template, and then second strand of cDNA was produced from the first strand of cDNA. Then, a 15-cycle-PCR reaction was used to finalize sequencing library of the quantified and purified double strands. Finally, Illumina HighSeq 4000 platform was used for paired-end sequencing. Then, Trinity was used to assemble high-quality clean reads (≥Q20) into unigenes with default parameters [90].

In this study, we identified all the putative protein coding sequences from aforementioned unigenes for further analysis. First, we blastx (ftp://ftp.ncbi.nlm.nih.gov/blast/executables/blast+/LATEST/) the unigenes sequence with the databases according to the priority order of Nr (http://www.ncbi.nlm.nih.gov), Swiss-Prot (http://www.expasy.ch/sprot), KEGG [18] and COG/KOG [91] (E-value cutoff level of <1E-5). Unigene sequences were aligned stepwise with protein sequences in the databases following the aforementioned order. The iterative process goes on unless a significant match is found in the current database. After that, we selected the highest rank protein from the blast alignment results to determine the coding region sequence of the unigenes, and then translated the coding sequence into amino acid sequence according to the standard codon table, so as to obtain the nucleic acid sequence (sequence direction 5′- > 3′) and amino acid sequence of the coding region of unigene. Finally, we predict coding region (nucleic acid sequence with direction 5′- > 3′) and corresponding protein sequence of unigenes that was aligned with above four databases by using program ESTScan [92]. Only those unigenes annotated by Nr, Swiss-Prot, KEGG and COG/KOG database or predicted coding region with program ESTScan were used for further analysis.

### Prediction of coding sequences and gene families

The unigenes annotated or predicted coding region by program ESTScan were pairwise aligned with Blastp software [93]. After the alignment, the pairwise unigenes with E-value lower than 1E-7 were regarded as homologous unigenes of intra- or inter-species. Then, unigenes, homologs (i.e. orthologs and paralogs) to each other, were classified into the same gene family by using OrthoMCL software v2.0.9 with default parameters (identity, coverage) [94]. The gene families with more than one unigene in any transcriptome of three blueberry species were regarded as multiple copy gene families, otherwise regarded as single copy gene family. The gene families with unigenes only from one blueberry species were defined as species-specific genes families. Meanwhile, homologous or unique gene or gene families were also obtained, and the number of gene families and corresponding unigenes number of three blueberry species were then counted and analyzed by Venny tool (https://bioinfogp.cnb.csic.es/tools/venny/).

### Phylogenetic tree construction with single copy unigenes

We clarified the genetic relationship of blueberry cultivars by constructing the molecular evolutionary tree based on single-copy homologous genes. First, we separately aligned all single copy unigenes families of three kinds of blueberry by program ClustalW (https://www.genome.jp/tools-bin/clustalw). Second, the multiple sequence alignment of all single-copy gene families were joined together to obtain the total single-copy gene sequence files for the construction of evolutionary tree. Finally, the phylogenetic tree was constructed by using the neighbor joining (NJ) method with 1000 iterations of MEGA software (https://www.megasoftware.net/), and the nucleotide substitute rate was regarded as the evolutionary unit of branch length in the phylogenetic tree.

### Ka/Ks test of single copy unigenes

We aligned protein sequences of single copy homologous unigenes of three kinds of blueberries by using the software muscle (http://www.drive5.com/muscle) implemented in MEGA10.1 software (https://www.megasoftware.net/). The aligned sequences were converted to corresponding nucleotide sequences by using RevTrans 1.4 [95]. Nonsynonymous substitution rate (Ka), synonymous substitution rate (Ks) of T1, T2, and T3, and Ka/Ks of all single copy homologous gene pairs (orthologs) of T1 versus T2, T1 versus T3, and T2 versus T3 were estimated by KaKs_Calculator Toolbox with the standard genetic code table (version 2.0) [7]. We filtered the single copy orthologs with no applicable values of Ka or Ks or Ks values > 0.1 (the benchmark of potential paralogs) as done by previous reports [74, 78, 96]. The validity of the Ka and Ks values were justified by Fisher’s exact test as described by Zhou et al. (2016) [82], and positively or negatively selected sites were allowed when P was < 0.05 and posterior probability was > 0.95 based on the results of Chi-square test as performed by Yang & Bielawski (2000) [97] and Hu et al. (2018) [79]. The low values of Ka/Ks for most of the non-synonymous mutations are harmful, and single-copy homologous gene make organisms less tolerant to harmful mutations because they have no copies. So, in order to find out the unigenes which underwent positive selection but might be neutralized (or covered) by a large number of harmful mutant sites, we defined single copy orthologs with Ka/Ks > 1 as strong positive selection, the Ka/Ks values between 0.5 and 1 as weak positive selection, and with Ka/Ks < 0.1 as negatively selected (purifying selection) according to previous reports [74, 79–81]. GO and KEGG enrichment analyses were performed by using GOseq [98] and KOBAS [99] of the orthologs sets with strong positive, weak positive and negative positive selection in three blueberry species, respectively. The *p*-value formula of the hypothesis test for significant enrichment was as follow:

$$ P=1-\sum \limits_{i=0}^{m-1}\frac{\left(\begin{array}{c}M\\ {}i\end{array}\right)\left(\begin{array}{c}N-M\\ {}n-i\end{array}\right)}{\left(\begin{array}{c}N\\ {}n\end{array}\right)} $$

Here, N is the number of genes with pathway (or GO) annotation in all unigenes; n is the number of species specific genes in N; M is the number of genes with a specific pathway (or GO) annotation in all unigenes; m is the number of genes with a specific pathway (or GO) annotation. The calculated *P*-value was further corrected by Bonferroni test, and the KEGG pathway (or GO term) with *p*-value < 0.05 was defined as the significantly enriched GO term (or GO term) for the species specific genes.

### Ks age distribution of duplicated unigene pairs (paralogs)

We identified the duplicated unigenes pairs (paralogs) of each blueberry crop from the coding sequence (cds) with 40% sequence similarity for at least 300 bp by the measure of discontinguous all-against-all MegaBLAST [100, 101]. We used the software muscle (http://www.drive5.com/muscle) to align the protein sequences of paralogs, and the aligned sequences were then converted to corresponding nucleotide sequences by using RevTrans 1.4 [61]. We calculated the Ks of all paralogs of each blueberry crop by using the KaKs_Calculator Toolbox with the standard genetic code table (version 2.0) [95]. To avoid the effects of Ks saturation that produced the multiplicative effects from multicopy gene families, we filtered the paralogs with Ks > 2 as done by Shi et al. [32]. We constructed the histogram of Ks density (age) distribution with bandwidth of 0.03 for each plot, and fitted a normal distribution curve with the Gaussian mixture model by mixtools program which built in R software [102]. We inferred the age of a duplication event by using the formula T_diversity_ = *Ks*/2r as described by Zhang et al. (2019b) [103]. We set the plant average *Ks*/year rate = 6.1 × 10^− 9^ for estimating the age of WGDs in blueberry species and median Ks value of each peak was used to determine the age of WGDs according to Lynch & Conery (2000) [104].

## Supplementary information

**Additional file 1: Table S1.** Statistics of unigenes undergone selection effect in three blueberry species.

**Additional file 2: Table S2.** Annotation summary of unigenes undergone positive selection.

**Additional file 3: Table S3.** GO enrichment summary of unigenes undergone positive selection in three blueberry species.

**Additional file 4: Table S4.** KEGG enrichment summary of unigenes undergone positive selection in three blueberry species.

## Data Availability

The datasets generated and/or analysed during the current study are available in the Genbank (Accessible ID: PRJNA511922) [NCBI] repository, Link: https://www.ncbi.nlm.nih.gov/search/all/?term=PRJNA511922, and the datasets used and/or analysed during the current study available from the corresponding author on reasonable request.

## Abbreviations

WGD: Whole genome duplication
T1: Rabbiteye blueberry
T2: Southern highbush blueberry
T3: Northern highbush blueberry
MYA: Million years ago
